# A Brief Introduction to Game Theory and its Potential Implications for the Economics of Orthotics & Prosthetics

**DOI:** 10.33137/cpoj.v4i2.36661

**Published:** 2021-09-21

**Authors:** G. Fiedler, A. Schikorra

**Affiliations:** 1 University of Pittsburgh, Department of Rehabilitation Science and Technology, Pittsburgh, USA.; 2 University of Pittsburgh, Department of Mathematics, Pittsburgh, USA.

**Keywords:** Healthcare Economics, Game Theory, Rehabilitation, Competitive Bidding

## Abstract

The economic viability of Orthotics & Prosthetics (O&P) service provision is an important concern for policy makers, patients, and practitioners. Against the background of limited funds that can be distributed for healthcare expenses overall, it is critical to identify the most cost-effective treatment options within and across disciplines, including surgical and pharmacological interventions. When those decisions are being negotiated, whether in the context of an individual case in the clinic or of general payer policies that allocate spending budgets, the O&P discipline is often perceived to be at a disadvantage due to its relatively young age, underdeveloped evidence base, and small economic clout as compared to other fields. Such asymmetrical negotiations have been the subject of economic theories and mathematical models, such as the “Game theory”, work on which has been awarded with several Nobel Prizes and other recognitions across the years. In this paper, we are introducing core concepts of this theory and discuss how they may be applied in negotiations on treatment approaches and reimbursement schedules with the goal to improve outcomes for the O&P profession.

## BACKGROUND

The costs of health care provision in the United States is estimated to exceed $4 trillion (T) in the year 2020.^1^ More than half of this amount is expended for hospital care ($1.3T) and physician and clinical services ($794B). Prescription drugs account for $358B, or about 9%. By comparison, only $62B, representing about 1.5% of the total, go into durable medical equipment of which orthotics and prosthetics (O&P) devices are a subsection.^1^ This distribution correlates with the size of the involved industries and their respective lobbying budgets. Data from the U.S. Bureau of Labor Statistics suggests that more than 750,000 physicians and more than 320,000 pharmacists were employed in 2019, which compares to a total of 10,000 O&P jobs nationwide.^2^ According to the Center for Responsive Politics, the pharmaceutical industry spent more than $300M on lobbying in 2020, followed by Hospitals/Nursing Homes with more than $100M.^3^ By comparison, the O&P Alliance, which is representing the interests of the main professional O&P organizations, had a lobbying budget of $60,000.^3^ Another measure of the disparity between O&P and other health professions is the amount of scientific evidence that informs their practice. The comparably small size of the O&P field corresponds with a small number of researchers working in this field and the associated number and quality of scientific publications.^4–6^

Overall, of course, this apparent imbalance is in large parts reflective of the patient populations tended to by the respective specialists. However, on the scale of individual patient cases there is, nonetheless, an overlap of competencies and a need for the entire health care team to collaboratively decide on the best treatment. Additional stakeholders include the patient and/or their family, as well as third-party payers, such as private and institutional health insurers or respective government agencies. If, in the example of a progressing orthopedic condition, it is to be decided whether conservative treatment or surgical intervention is called for, a variety of explicit and implicit interests of these stakeholders need to be reconciled. The patient is likely to prioritize the subjectively most promising and convenient treatment irrespective of the costs, whereas the insurer is interested in the most cost-effective treatment, which ideally includes a consideration of long-term costs.

For the healthcare professionals, the eventual decision will have obvious economic implications as well. The surgeon's recommendation, by nature, may be biased toward surgical treatment, just as the orthotist's recommendation may favor bracing. A similar dynamic applies when insurance coverage and reimbursement schedules are being established and/or revised.

The process of arriving at the decision can by some definition be considered a negotiation. With the comparative prestige of their academic degree and the amount of citable evidence to support their case likely not on the side of the O&P practitioners in this scenario, they may be at a disadvantage in such a negotiation.

A different problem is that of competing against another provider. There may be various examples of this occurring in everyday practice but an instance with especially high stakes is the dreaded competitive bidding process that is frequently utilized, or at least proposed, by institutional payers, including the Centers for Medicare and Medicaid Services.^7^ In some such scenarios, O&P providers may have to contend against other bidders from outside the actual profession. If those competitors have a different cost structure, for instance, by being subject to different education and licensing requirements or by having lower local costs for labor and parts, the bidding process is unlikely to be effective in optimizing the economics of the contract at stake. Instead, it may happen that the expert O&P provider is priced out and the winning bid does not cover the costs of providing quality care, to the detriment of the patient, the provider, and ultimately, the insurer as well.

These scenarios pose the question if there are strategies to optimize the outcomes under a given (or assumed) set of circumstances. An answer to that question may be offered by game theory, chiefly a set of mathematical models to describe and predict negotiation dynamics, which has been recognized as an important concept in economics.^8,9^

### The mathematical basis of game theory

For simplicity we focus here on so-called non-cooperative games of, for example, two decision makers (called the players). Our game is pretty short and simple: each of the players makes a decision and receives a payout (or has to pay) according to some rules.

Take, as an example, two doctors who have to decide which treatment to prescribe for a certain type of illness. Let us say, Treatment 1 costs $900, and Treatment 2 costs $1,000. If both doctors agree that Treatment 2 is better, they will likely (in the long run) receive both 50% of the patients to treat. If, however, one of the doctors decides to prescribe Treatment 1 (even though Treatment 2 might be better), the insurance has – all else being equal – an incentive to choose the doctor that is cheaper. Knowing this, both doctors have an incentive to prescribe Treatment 1 (even though it may be not the optimal treatment) for the fear of losing all revenue. So, according to this model both doctors are coerced to recommend the cheaper treatment-regardless of the qualitative advantages of Treatment 2 (Table 1).

**Table 1:** Payout structure for each possible combination of prescription decisions.

		Doctor 2's prescription			
		Treatment 1 ($900)		Treatment 2 ($1000)	
		Doctor 1 gets	Doctor 2 gets	Doctor 1 gets	Doctor 2 gets
**Doctor 1‘s prescription**	Treatment 1	50% of 900	50% of 900	100% of 900	0% of 1000
	Treatment 2	0% of 1000	100% of 900	50% of 1000	50% of 1000

In mathematical terms the choice for Treatment 1 is called the Nash equilibrium: it is a choice where, for each player, the other party cannot improve their outcome by changing strategies. The problem that the Nash equilibrium may not be the optimal outcome for the participants is called prisoners’ dilemma of game theory. And this effect has been observed in many real-world situations (e.g., in social psychology or drug cartel formation).^10,11^

The mathematical models (and summary tables) for this sort of real-life situation quickly become much more complex if additional parameters are being considered. In the simplified example of a “Cardinal game” above, for instance, the insurance is making decisions solely by price tag, which may not be entirely realistic. If the outcomes are on an ordinal scale rather than binary as in this example, or if there are additional differences between providers (e.g., qualifications, seniority, etc.) that affect the decision making, the model needs to be expanded and the additional assumptions need to be codified to allow for calculation of the Nash equilibrium.

### Implications for negotiations in the O&P realm

It may not be immediately obvious how the dynamics of a “prisoners' dilemma” could apply to the realm of O&P related economics. Indeed, most of the typical scenarios with which stakeholders in this field are confronted can be explained (and possibly solved) as a simple optimization problem. For instance, a negotiation with a payer (health insurance) about whether to give a transfemoral prosthesis patient a conventional hydraulic knee joint or a microprocessor-controlled knee can be reduced to a fairly straightforward balancing of costs and benefits. The cost differential is well known, and the risks of, say, accidental fall(s) that are detrimental to quality of life and follow-on health care cost savings^12,13^ can be reasonably approximated using historical data and a probabilistic distribution curve. These factors will allow the recommendation to utilize either knee type at a specific ratio to optimize outcomes for all involved (at least on average).

A more suitable application may be found in the competitive bidding process. An aspect of this process in the O&P field is the, theoretically, unlimited number of bidders (i.e., players), as well as a large number of contractual terms and performance criteria, making it difficult to model as a Cardinal game, which has a specific payoff function. Instead, a more complex ordinal game theory approach may be appropriate. Recent work^14^ suggests that such a model and associated optimization algorithm is applicable to general variants of Public-Private Partnerships, claiming that, among other things, “(1) it can handle any number of private sector players and … performance criteria, (2) it determines a single ranking of proposals …, [and] (3) it can be used by the private sector players … to assist with the choice of bidding strategies…”.^14^ Given the inevitable differences between the specific scenarios considered in this research and the real-world process of entering a bid in, say, the CMS Durable Medical Equipment, Prosthetics, Orthotics, and Supplies (DMEPOS) Competitive Bidding program, more work to validate and refine the model is likely required. This work will include a discipline-specific ranking of the “strategy profile-induced outcomes”, to include things such as long-term health outcomes, processing efficiency, and sustainability of the provider pool. Still, it offers an interesting approach to maximizing the limited leverage wielded by O&P providers in the competitive bidding process and to optimize the bid evaluation and contracting process by the payer.

## CALL TO ACTION

The long-term health and sustainability of our industry requires a mitigation of the inherent disadvantage that small-size businesses, such as typical O&P providers, have in competitive bidding and contract negotiations. We believe that modern game theory is a tool that can help address this issue, and we call on the large professional organizations that represent and cater to those businesses (e.g., the American Orthotic & Prosthetic Association and the O&P Alliance) to explore ways to take advantage of this powerful tool. This effort could take the shape of adding game theory content to business training offerings, or providing grant support for the development of industry-specific game theory models.

## DECLARATION OF CONFLICTING INTERESTS

The authors declare no conflicts of interest related to this work.

## SOURCES OF SUPPORT

This work was partially supported by Simons foundation grant no 579261 and by NSF Career DMS-2044898
